# Development of a Once-Daily Modified-Release Formulation for the Short Half-Life RIPK1 Inhibitor GSK2982772 using DiffCORE Technology

**DOI:** 10.1007/s11095-021-03124-7

**Published:** 2022-01-05

**Authors:** Debra Tompson, Mark Whitaker, Rennan Pan, Geoffrey Johnson, Teresa Fuller, Vanessa Zann, Litza McKenzie, Kathy Abbott-Banner, Simon Hawkins, Marcy Powell

**Affiliations:** 1 Clinical Pharmacology Modelling and Simulation, Medicines Research Centre, GlaxoSmithKline, Gunnels Wood Road, Stevenage, SG1 2NY Hertfordshire UK; 2 Medicine Process Delivery, GlaxoSmithKline, Dave Jack Medicines Development Centre, Park Road, SG12 0DP Hertfordshire, UK; 3 Pharmaceutical Development, GlaxoSmithKline, 1250 S. Collegeville Road, Collegeville, Pennsylvania 19426 USA; 4 Development Biostatistics, GlaxoSmithKline, 1250 S. Collegeville Road, Pennsylvania 19426 Collegeville, USA; 5 Global Clinical Sciences and Delivery, Medicines Research Centre, GlaxoSmithKline, Gunnels Wood Road, Stevenage, SG1 2NY Hertfordshire UK; 6 Quotient Sciences Limited, Mere Way, Ruddington, NG11 6JS Nottingham UK; 7 Discovery Medicine, GlaxoSmithKline, 980 Great West Road, Brentford, TW8 9GS Middlesex UK; 8 Safety and Medical Governance, GlaxoSmithKline, 5 Moore Drive, Research Triangle Park, North Carolina, 27709-3398 USA

**Keywords:** DiffCORE, GSK2982772, modified release, RIPK1, translational pharmaceutics

## Abstract

**Purpose:**

GSK2982772 is a selective inhibitor of receptor-interacting protein kinase-1 (RIPK1) with a short 2- to 3-h half-life. In a previous modified-release (MR) study, a matrix monolithic formulation (80% GSK2982772 released over 12 h) provided a once-daily (QD) pharmacokinetic (PK) profile in the fasted state; however, it was susceptible to food effects. The current study evaluated the safety and PK of MR formulations using GSK proprietary DiffCORE™ technology.

**Methods:**

Part A evaluated PK following single-dose (240 mg) fasted and fed (high-fat meal) administration of three DiffCORE MR formulations within pre-defined *in vitro* extremes of 80% GSK2982772 released over 12 h (MR-12 h) to 80% GSK2982772 released over 18 h (MR-18 h) versus an immediate-release formulation. Part B evaluated MR-16 h (120–960 mg) in different prandial states.

**Results:**

Pharmacokinetic profiles for all MR formulations and doses tested in the fasted and fed states were consistent with QD dosing.

**Conclusions:**

The DiffCORE technology overcame the food effect vulnerability observed with the matrix monolithic formulation. The MR-16 h formulation was selected for further clinical development as a QD dosing regimen (NCT03649412 September 26, 2018).

**Supplementary Information:**

The online version contains supplementary material available at 10.1007/s11095-021-03124-7.

## INTRODUCTION

GSK2982772 is a highly selective, receptor-interacting protein-1 kinase (RIPK1) inhibitor being developed for the treatment of plaque psoriasis and other immune-mediated inflammatory diseases. RIPK1 has dual roles as a kinase and a scaffolding protein. Through both functions, it acts as a key mediator of cell death and inflammation downstream of numerous pathways and signaling receptors, including the tumor necrosis factor (TNF) family of cytokines (1, 2). Recent work has demonstrated that RIPK1 activity can regulate TNF-mediated necroptosis and apoptosis (2–4). RIPK1 also facilitates TNF-mediated classical apoptosis and nuclear factor kappa-light-chain-enhancer of activated B cells (NFκβ) signaling through its scaffolding function (2, 5, 6). Inhibitors of RIPK1 activity are therefore being investigated in diseases linked to TNF activation, including plaque psoriasis and other inflammatory diseases (1, 7, 8).

GSK2982772 is considered a Biopharmaceutics Classification System (BCS) class 2 drug substance characterized by high passive permeability (human Peff [effective permeability] > 2 × 10^–4^ cm/s), and low solubility (0.1 mg/mL) (9, 10). Despite the low solubility, when GSK2982772 is administered either as standard immediate-release (IR) tablets or powder in capsules, there is no evidence of dissolution rate-limiting absorption up to a dose of 240 mg (11). Median time to maximum concentration (T_max_) is approximately 2 h post-dose (8). After attainment of maximum plasma concentration (C_max_), concentrations decline rapidly until approximately 12 h post-dose. Most systemic exposure is associated with an initial 2–3 h half-life (t_½_) followed by a slower terminal phase t_½_ of approximately 5 − 6 h. As a result, initial clinical trials conducted with GSK2982772 have used twice-daily (BID) and thrice-daily (TID) regimens with IR tablets (60 mg) (7, 12).

For chronic inflammatory conditions, a once-daily (QD) dosing option would offer greater convenience, potentially improve compliance and therapeutic outcome, and offer a flatter concentration–time profile with lower peak-to-trough concentrations compared with the IR formulation. A previous study evaluated matrix-based MR formulations of GSK2982772 administered as either minitablets (MT) or matrix monolithic (MM) tablets using two *in vitro* dissolution rates: a) 80% GSK2982772 released over 8 h, and b) 80% GSK2982772 released over 12 h (13). This previous study showed that a QD pharmacokinetic (PK) profile could be achieved in the fasted state with the slower *in vitro* dissolution rate of 80% release over 12 h (MT-12 h and MM 12 h). However, the matrix-based formulation was susceptible to a food effect when administered with a high-fat meal, resulting in most of the exposure to GSK2982772 occurring within the first 12 h of dosing, which was not considered to be optimal for QD dosing.

For a low-solubility drug such as GSK2982772, the primary release mechanism from a matrix-based tablet formulation is by erosion. Due to the short retention time of a tablet in the fasted stomach (~ 0.5 h) (14), the mechanical stress experienced as a result of stomach contractions is relatively low as the formulation matrix is minimally hydrated in this time and is therefore likely to pass through into the small intestine before substantial mechanical erosion occurs. However, when a matrix MR tablet is administered with a high-fat meal, the tablet is retained in the stomach for 4–6 h and the digestive mechanical stress increases the extent of erosion of the matrix tablets, thereby increasing gastric drug release (10, 15). When the stomach empties, the dissolved drug then becomes available for absorption. In order to reduce the possibility of food effects with high-fat meals, the current study evaluated MR formulations utilizing GSK proprietary DiffCORE™ technology, which has previously shown robustness against food effects (e.g., Lamictal XR) (16). DiffCORE™ combines traditional polymer matrix with an outer enteric coat with apertures mechanically drilled into the coat. The enteric coating is insoluble in the low pH gastric environment, but dissolves in the high pH intestinal environment. While the DiffCORE formulation is in the stomach, the enteric coating protects the tablet core from digestive mechanical stress, whilst the drilled apertures allow some drug release.

This study was designed to determine the optimum release rate of GSK2982772 for a once daily dosing regimen. In addition, the effect of co-administration of food (high-fat or standard meal) with the MR formulations on the PK of GSK2982772 was evaluated, and a preliminary assessment of PK linearity over a dose range of 120 to 960 mg was assessed.

## MATERIALS AND METHODS

**Fig. 1:**
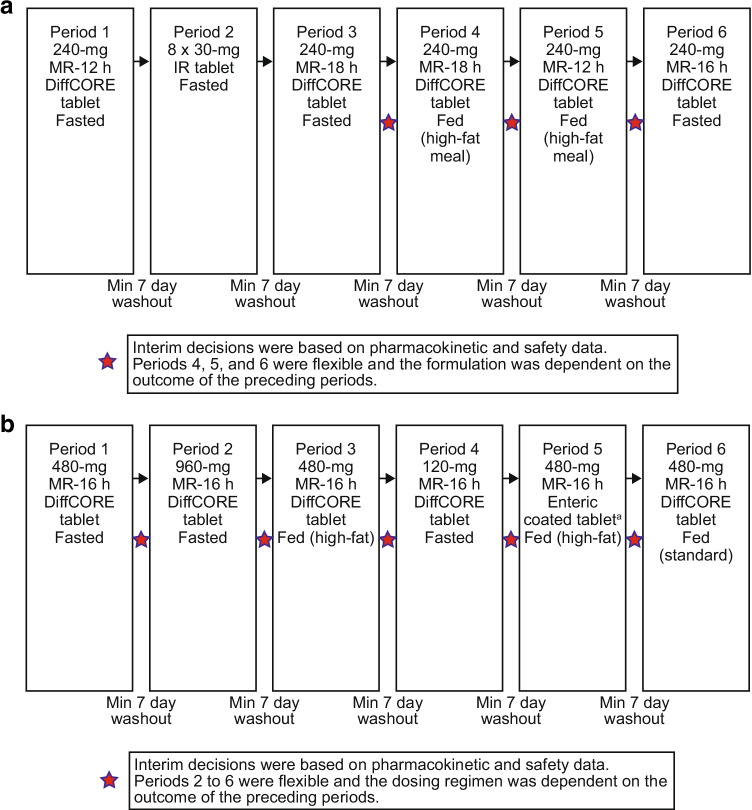
Study design. **(a)** Part A Formulation Optimization and Food Effect **(b)** Part B Tablet Strength and Food Effect. h, hours; IR, intermediate release; MR-12 h, modified release with 80% release at 12 h; MR-16 h, modified release with 80% release at 16 h; MR-18 h, modified release with 80% release at 18 h. ^a^Tablet manufactured without apertures (non-drilled).

An integrated good manufacturing practice (GMP) and clinical testing platform (Translational Pharmaceutics) (17) was used for this study, which allows formulation optimisation (e.g., formulation release rate, dose level, and prandial state) during a clinical study based on emerging clinical PK data (Fig. 1). The formulation consisted of two hydroxypropyl methylcellulose (HPMC) polymers. The total polymer weight relative to the total GSK2982772 weight was fixed across formulations and dose strengths. Adjusting the ratio of polymers allowed for a range of in vitro release rates to be evaluated within pre-defined extremes. Drug release was determined using a U.S. Pharmacopeia (USP) II paddle method. The extremes of release rates ranged from 80% GSK2982772 release over 12 h (MR-12 h) to 80% GSK2982772 release over 18 h (MR-18 h). The MR-12 h formulation corresponds to the slowest release rate used in the previous prototype matrix MR study, which provided a superior QD PK profile to the 80% release over 8 h matrix formulation.

This was a 2-part, non-randomized, open-label study. Part A was a 6-period, 6-way fixed sequence design assessing the PK profile of MR formulations of GSK2982772 relative to an IR reference tablet formulation at a fixed-dose strength (240 mg) in healthy subjects. Three MR formulations with different release rates were evaluated following single-dose administration in the fasted or fed state (Fig. 1a). Periods 1, 2, and 3 were defined a priori and evaluated MR-12 h, IR, and MR-18 h respectively, in the fasted state. Interim decisions based on the review of safety and PK data after Periods 3, 4, and 5 determined the formulation and prandial status to be investigated in the next period. In Periods 4 and 5, MR-18 h and MR-12 h, respectively, were dosed following a high-fat meal. In Period 6, MR-16 h was dosed in the fasted state. Based on the reviews of PK and safety data following Part A, the MR-16 h regimen was selected for Part B.

Part B was an open-label, non-randomized, up to 7-period fixed-sequence study design evaluating the PK profile for MR-16 h formulation at differing dose levels and prandial states (meal content and meal timing) (Fig. 1b). The highest dose of 960 mg used in Part B was selected based on the bioavailability of the MR-16 h formulation relative to the IR tablet from Part A and the exposure of GSK2982772 achieved in a high-dose PK study in which doses of IR tablets up to 240 mg TID were administered (11). An interim review followed each period to determine the dose level and prandial status for each subsequent period. Periods 1 and 2 evaluated MR-16 h 480 and 960 mg, respectively, in the fasted state. Period 3 evaluated 480 mg (high-fat meal); Period 4, 120 mg (fasted); and Period 6, 480 mg (standard meal). A 480-mg enteric-coated formulation with the same core matrix as MR-16 h was evaluated in Period 5 (high-fat meal). Optional Period 7 (Part B) was not conducted.

The study was approved by the Wales Research Ethics Committee 2 (Castlebridge 4, 15–19 Cowbridge Road East, Cardiff, UK, CF11-9AB) and was conducted according to the recommendations of Good Clinical Practice and the 1964 Declaration of Helsinki and its later amendments. All subjects provided written informed consent to participate in the study.

### Study Treatment

Inpatient periods for MR regimens consisted of 4 days and 3 nights, and inpatient stays for the IR regimen (Period 2 of Part A) were 3 days and 2 nights. Each treatment period was separated by a minimum of 7 days of washout, and a follow-up visit occurred 7 to 9 days after the last study treatment. For fasted regimens, subjects were dosed in the morning following a 10-h overnight fast. In the fed state, subjects were dosed 30 min after the meal was served and at least 90% of the meal had to be consumed for dosing to proceed. The standard meal consisted of 50 g of cereal, 200 mL semi-skimmed milk, 1 croissant or bread roll, and 1 portion of jam. The high-fat meal consisted of 1 hash brown, 2 strips of bacon, 1 small fried egg, 2 slices of buttered white bread, and 240 mL whole milk, with caloric value and fat content (882 kcal, with 54 g providing 486 kcal [55%] from fat) consistent with US Food and Drug Administration [FDA] test meal recommendations (18).

### Subjects

Healthy male and female subjects 18 − 65 years of age, with body weight ≥ 50 kg and body mass index of 19.0 − 32.0 kg/m^2^ were eligible. Health status was determined by medical history, physical examination, laboratory tests, and cardiac monitoring.

### PK Sampling

Pharmacokinetic blood samples were collected pre-dose and every 2 h post-dose up to 48 h following dosing of MR formulations. For the IR formulation, samples were collected pre-dose, and post-dose at 20 min, 40 min, 1 h, 1.5 h, and 2 h, and every hour thereafter until 6 h. Between 6 and 12 h samples were collected every 2 h, with a final sample at 24 h post-dose. Blood samples were centrifuged within 30 min of collection, and supernatant plasma was frozen at –20˚C. Plasma samples were shipped frozen on dry ice to Covance Laboratories (Harrogate, UK), and GSK2982772 concentrations were determined using validated analytical methods (according to GSK standard operating procedures and bioanalytical method validation guidelines from the FDA and European Medicines Agency) (19, 20). Human plasma samples were analysed for GSK2982772 using a validated analytical method based on protein precipitation, followed by HPLC/MS/MS analysis. The lower limit of quantification was 1 ng/mL using a 25-µL aliquot of human plasma, with a higher limit of quantification of 1000 ng/mL.

### PK Parameters

Pharmacokinetic parameters were calculated by standard non-compartmental analysis using WinNonlin v8.0. The following PK parameters were determined from the plasma concentration–time data for each regimen: maximum observed plasma concentration (C_max_); time to C_max_ (T_max_); observed concentration at 24 h post-dose (C_24h_); relative bioavailability of test formulation vs reference formulation (Frel_formulation_) based on area under the curve (AUC) from zero to infinity (AUC_[0-inf]_) (or AUC_[0-t]_ when AUC_[0-inf]_ could not be derived); and relative bioavailability of fed vs fasted (Frel_FE_) based on C_max_ and AUC. In addition (for Part B only), C_max_/dose and AUC/dose_(0–24)_ were derived to evaluate dose linearity and relative bioavailability of enteric-coated (Frel_enteric_) to DiffCORE was based on AUC and C_max_.

### Statistical Analyses

Descriptive statistics were calculated by the study treatment group for all PK concentrations over time and for the derived PK parameters. In addition, for log-transformed PK parameter variables (AUC and C_max_), geometric mean, 95% confidence interval (CI), and between-participant coefficient of variation (%CV_b_) (100 * √(exp(SD^2^) − 1)) were provided, where the SD was the standard deviation of log-transformed data. Estimation of geometric mean ratios was performed using mixed models.

### Safety

Safety assessments included adverse event (AE) monitoring, vital signs, electrocardiograms (ECGs), physical examination, and clinical laboratory tests. Laboratory results were available prior to dosing on day 1 and assessments were repeated on day 2 of each treatment period.

## RESULTS

### Subjects

A total of 17 subjects were enrolled in Part A, including one subject to replace a subject who was discontinued at the discretion of the investigator in Period 1. In addition, two subjects withdrew because of an AE (one with gastroenteritis and one with transaminases increased), and one subject withdrew consent, for a total of 4 who discontinued Part A early. In Part A, 10 (59%) subjects were male and 16 (94%) were Caucasian/European; one was Arabic/North African. Baseline characteristics for subjects who participated in the study can be found in Online Resource I.

In Part B, 16 subjects were enrolled and 14 (88%) completed the study. One subject was withdrawn due to depressed mood and one because of actinic keratosis. In Part B, 10 (63%) subjects were male, 13 (81%) subjects were Caucasian/European, one was African American, one was of central/south Asian heritage, and one was of mixed race.

### Safety

In Part A, no drug-related AEs were reported and no serious AEs were reported. There was no clear trend in the occurrence of AEs by treatment group (Online Resource II). Only headache (*n* = 4: 2 subjects in the 240-mg MR-12 h fasted group and 2 subjects in the 240-mg MR-18 h fed group) and upper respiratory tract infection (2 subjects in the 240-mg MR-16 h fasted group) were reported by more than one subject in any treatment group. One subject in Part A was withdrawn due to a mild AE of gastroenteritis, which resolved spontaneously within 2 days. Two moderate AEs (one subject with nasopharyngitis, 240-mg MR-12 h fed [high-fat] group; one subject with upper respiratory infection, 240-mg MR-16 h fasted group) occurred. One subject was withdrawn (240-mg MR-18 h fed [high-fat] group) after receiving 4 doses of GSK2982772 due to a severe AE of transaminase increased with concomitant significant elevation of creatine kinase, which was judged to be related to strenuous exercise. All liver function parameters had resolved when the subject returned for follow-up approximately 3 weeks later. All other AEs were of mild intensity.

Similar to Part A, there were no apparent trends in the occurrence of AEs (Online Resource II). Headache was the most common AE, reported by 3 (19%), 1 (7%), 1 (7%), and 2 (13%) subjects in the 480-mg MR-16 h fasted, 480-mg MR-16 h fed (standard), 480-mg enteric-coated MR-16 h fed (high-fat), and 960-mg MR-16 h fasted treatment groups, respectively. Drug-related AEs included headache (2 [13%]) and vomiting (1 [7%]), which were reported following administration of the 960-mg MR-16 h fasted regimen. A mild AE of nightmare, reported by 1 (6%) subject in the 480-mg MR-16 h fasted group, was also judged to be drug related. One subject in the 480-mg MR-16 h fasted group was withdrawn due to an intermittent mild AE of depressed mood. The subject had a history of depression, which had resolved more than 10 years prior to the study. Another subject (480-mg MR-16 h fed [high-fat] group) was withdrawn due to a nonhealing lesion on the forehead diagnosed as actinic keratosis. Neither AE leading to withdrawal was deemed to be related to GSK2982772. No serious AEs occurred, and no AE was judged to be of moderate or severe intensity.

With the exception of one report of severe transaminase elevation (Part A), no clinically significant laboratory results were reported for any individual subject. There were no notable trends in laboratory parameters. No clinically important changes were reported for laboratory assessments, vital signs, or ECG findings in either part of the study.

### Non-compartmental Pharmacokinetic Analyses

#### Part A

Decisions regarding formulation release rate and prandial state for Part A were made as follows:

- From Periods 1–3, both 240-mg MR-12 h and MR-18 h DiffCORE formulations administered in the fasted state had PK profiles consistent with the desired QD dosing. Overall, the MR-18 h formulation had a flatter PK profile than the MR-12 h formulation due to a lower C_max_ and higher C_24h_. Following review of PK data from Periods 1–3, it was decided to evaluate the impact of co-administering MR-18 h and MR-12 h with a high-fat meal in Periods 4 and 5, respectively.
- In Periods 4 and 5, the PK performance for both MR-18 h and MR-12 h formulation following administration with a high-fat meal was similar to administration in the fasted state. Based on these results, it was decided for Period 6 to evaluate a formulation with a dissolution rate of 80% over 16 h (MR-16 h), which was close to the MR-18 h formulation which had a flatter GSK29872772 concentration–time profile, but would allow some formulation flexibility away from the slowest release rate in the design space.
- In Period 6, the MR-16 formulation had a similar PK profile to the MR-18 h formulation when administered in the fasted state. The MR-16 h formulation was selected for evaluation in Part B. Since both MR-12 h and MR-18 h showed similar PK when administered in the fasted and fed (high-fat) states, it was assumed that this would be applicable to MR-16 h.

**Fig. 2:**
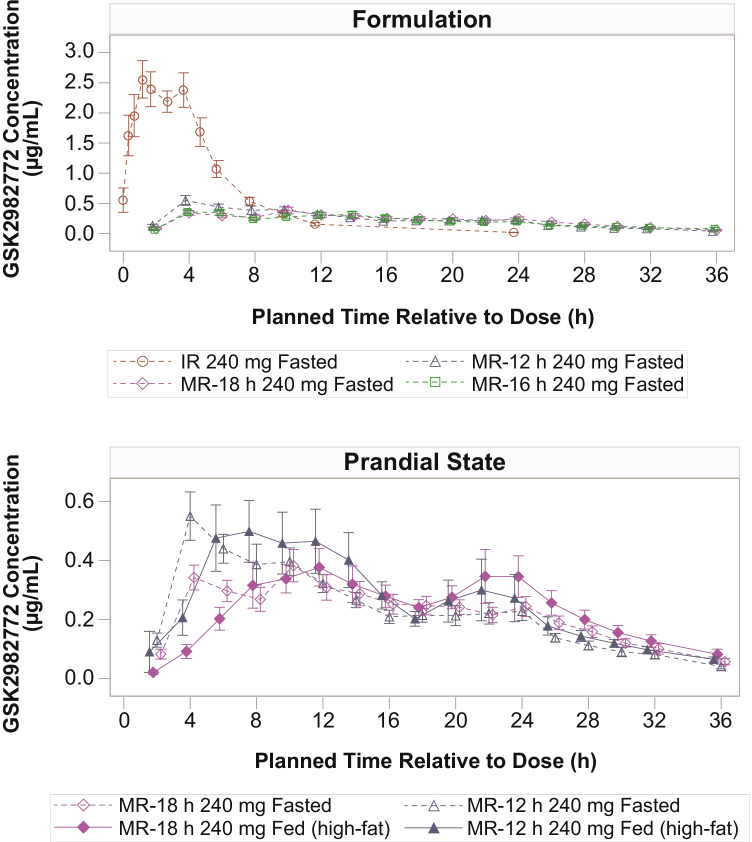
Mean plasma GSK2982772 concentration–time plots by formulation (fasted) and prandial state (Part A) for 240-mg dose. h, hours; IR, intermediate release; MR-12 h, modified release with 80% release at 12 h; MR-16 h, modified release with 80% release at 16 h; MR-18 h, modified release with 80% release at 18 h. Error bars represent ± 1 standard error.

Overall, following administration of 240-mg MR-12 h, MR-16 h, and MR-18 h DiffCORE formulations in the fasted state, the concentration–time profiles of GSK2982772 demonstrated prolonged absorption characterized by a delayed T_max_ and reduced C_max_ and AUC compared with the IR formulation (Fig. 2). The time to maximum concentration increased as the rate of dissolution became slower. Median T_max_ increased from 2 h for the IR formulation to 5, 6, and 10 h post-dose for the fasted MR-12 h, MR-16 h, and MR-18 h DiffCORE formulations, respectively (Online Resource III). Geometric mean C_max_ values for MR-12 h, MR-16 h, and MR-18 h DiffCORE formulations were 22%, 14%, and 17% that of IR C_max_, respectively. Based on AUC_(0-inf)_, the bioavailability for MR-12 h, MR-16 h, and MR-18 h DiffCORE formulations relative to IR was similar across the formulations (55%, 48%, and 58%, respectively) (Table I). Geometric mean C_24h_ values for MR-12 h, MR-16 h, and MR-18 h DiffCORE formulations were approximately 11- to 14-fold higher than for the IR formulation (Table I).

**Table I:** Summary of statistical analyses of plasma GSK2982772 PK parameters for DiffCORE MR formulations versus IR (geometric mean ratios [90% CI]) (Part A)

240-mg MR Formulation Bioavailability vs 240-mg IR in the Fasted State			
	MR-12 h vs IR (*N* = 16)	MR-16 h vs IR (*N* = 12)	MR-18 h vs IR (*N* = 16)
C_max_	0.223 (0.188–0.265)	0.142 (0.118–0.171)	0.166 (0.140–0.197)
T_max_^a^	3.00 (2.50–4.50)	4.50 (3.00–8.00)	6.53 (3.00–8.50)
AUC_(0-inf)_	0.547 (0.476–0.628)	0.483 (0.410–0.570)	0.577 (0.506–0.658)
C_24h_	12.7 (8.85–18.1)	10.5 (7.13–15.5)	13.6 (9.52–19.4)
Food Effect Relative Bioavailability, Fed (High-Fat) vs Fasted			
	MR-12 h (*N* = 12)	MR-18 h (*N* = 16)	
C_max_	1.12 (0.929–1.354)	1.29 (1.09–1.53)	
T_max_^a^	2.00 (0.00–6.00)	4.00 (− 0.03 to 6.00)	
AUC_(0-inf)_	1.14 (0.955–1.35)	1.03 (0.888–1.19)	
AUC_(0-t)_	1.08 (0.952–1.22)	1.07 (0.957–1.20)	

CI, confidence interval; h, hours; IR, intermediate release; MR-12 h, modified release with 80% release at 12 h; MR-16 h, modified release with 80% release at 16 h; MR-18 h, modified release with 80% release at 18 h

^a^Median difference, 90% CI

**Fig. 3:**
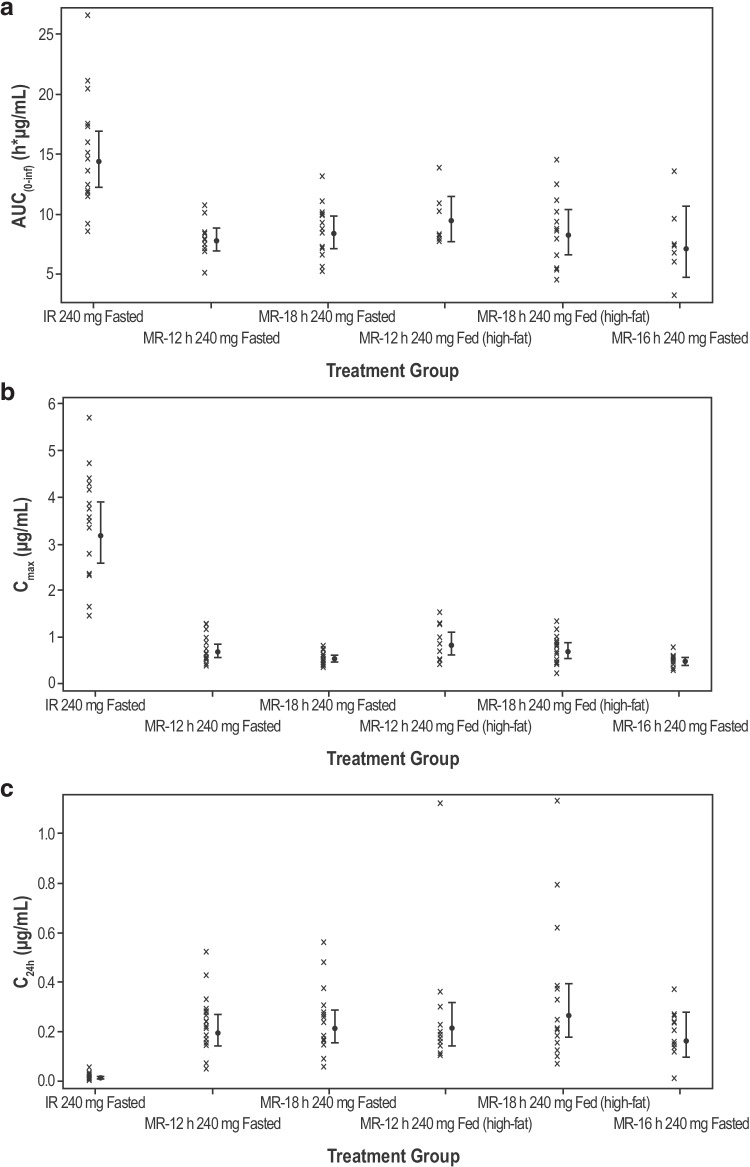
Individual subject (geometric mean and 95% CI) plasma PK parameters by formulation and prandial state (Part A). **(a)** Plasma AUC_(0-inf)_ (h*µg/mL). **(b)** Plasma C_max_ (µg/mL). **(c)** Plasma C24h (µg/mL) CI, confidence interval; h, hours; IR, intermediate release; MR-12 h, modified release with 80% release at 12 h; MR-16 h, modified release with 80% release at 16 h; MR-18 h, modified release with 80% release at 18 h.

Following 240-mg DiffCORE MR dosing with a high-fat meal, T_max_ was delayed compared with the fasted state (Fig. 2). There was a small increase in C_max_ (MR-12 h, 12%; MR-18 h, 29%) compared with the fasted state, but no notable differences in AUC **(Table ****I**** and Fig. ****3****).**

#### Part B

Decisions regarding dose level and prandial state for Part B were made as follows:

- In Period 1, 480 mg MR-16 h was evaluated in the fasted state. The PK of GSK2982772 appeared linear between the 480-mg MR-16 h (fasted) formulation in Part B and the 240-mg MR-16 h (fasted) formulation from Part A, indicating no solubility rate-limiting absorption at the 480-mg dose. For Period 2, the MR-16 h dose was increased to 960 mg (administered as 2 × 480 mg) to further evaluate the PK linearity. The 960-mg dose was selected as this represents the highest dose likely to be taken into future phase 2 dose-ranging studies.
- In Period 2, following administration of 960-mg MR-16 h in the fasted state, there was an approximate linear increase in exposure for C_max_ and AUC between 480 and 960 mg. Since there was no apparent solubility rate-limiting absorption of GSK2982772 in the fasted state, the impact of administering 480-mg MR-16 h with a high-fat meal was evaluated in Period 3. Even though a food effect was not observed for the 240-mg tablet, it was hypothesized that larger tablets may be retained in the stomach for longer and therefore food may have a greater effect on PK (14).
- In Period 3, administration of 480 mg MR-16 h with a high-fat meal had a greater effect for the 480-mg tablet strength than for the 240-mg tablet strength but still maintained GSK2982772 concentration–time profiles consistent with QD dosing.
- Following review of the data from Periods 1 to 3, a dose of 120 mg in the fasted state, near the lower end of the dose range likely to be assessed in a future phase 2 dose-ranging study, would be evaluated in Period 4.
- For Period 5, the effect of co-administering a high-fat meal with a 480-mg fully enteric-coated tablet with an MR-16 h core and the impact on the PK without the drilled apertures of the DiffCORE formulation was evaluated.
- Period 6 evaluated 480 mg DiffCORE MR-16 h following a standard meal to see if administration of a lighter meal reduced the food impact.

In Part B, dose proportionality of the MR-16 h DiffCORE formulation was assessed for the 120-, 480-, and 960-mg doses in the fasted state. There were dose-proportional increases in exposure for C_max_ and AUC between 480 and 960 mg (Online Resource IV and Table II). However, there was a less than dose-proportional increase in exposure between 120 and 480 mg. For the 4-fold increase in dose between 120 and 480 mg, there was a 2.12-fold increase in C_max_ and a 2.48-fold increase in AUC_(0-inf)_ (Online Resource IV and Table II). For 480 mg MR-16 h in the fasted state, median T_max_ was 6 h and increased to 9 h when administered with a standard meal and 12 h with high-fat meal.

**Table II:** Summary of statistical analyses of plasma GSK2982772 PK parameters assessing DiffCORE MR dose linearity, food effect and enteric-coated MR versus DiffCORE MR (geometric mean ratios [90% CI]) (Part B)

Assessing Dose Linearity of MR-16 h Fasted		
	960 vs 480 mg (*N* = 15)	480 vs 120 mg (*N* = 16)
C_max_	1.60 (1.28–2.00)	2.12 (1.70–2.64)
T_max_^a^	− 0.05 (− 4.00 to 0.00)	0.00 (− 4.00 to 2.00)
AUC_(0-inf)_	2.14 (1.71–2.68)	2.48 (2.02–3.05)
C_24h_	2.17 (1.26–3.74)	7.74 (4.50–13.3)
Assessing Food Effect Relative Bioavailability for 480 mg MR-16 h		
	Fed (High-Fat) vs Fasted (*N* = 15)	Fed (Standard) vs Fasted (*N* = 14)
C_max_	1.73 (1.39–2.16)	1.02 (0.816–1.28)
T_max_^a^	6.00 (2.00–12.1)	2.00 (− 0.07 to 4.17)
AUC_(0-inf)_	1.42 (1.13–1.77)	0.971 (0.785–1.20)
AUC_(0-t)_	1.30 (1.09–1.54)	0.898 (0.754–1.07)
480-mg Enteric-Coated MR-16 h Fed (High-Fat) vs 480-mg DiffCORE MR-16 h Fed (High-Fat)		
	Enteric-Coated MR vs DiffCORE MR (*N* = 15)	
C_max_	0.944 (0.752–1.19)	
T_max_^a^	2.00 (− 1.98 to 9.98)	
AUC_(0-inf)_	0.975 (0.768–1.24)	
AUC_(0-t)_	0.890 (0.746–1.06)	
C_24h_	1.80 (1.03–3.15)	

CI, confidence interval; h, hours; MR-16 h, modified release with 80% release at 16 h

^a^Median difference, 90% CI

**Fig. 4:**
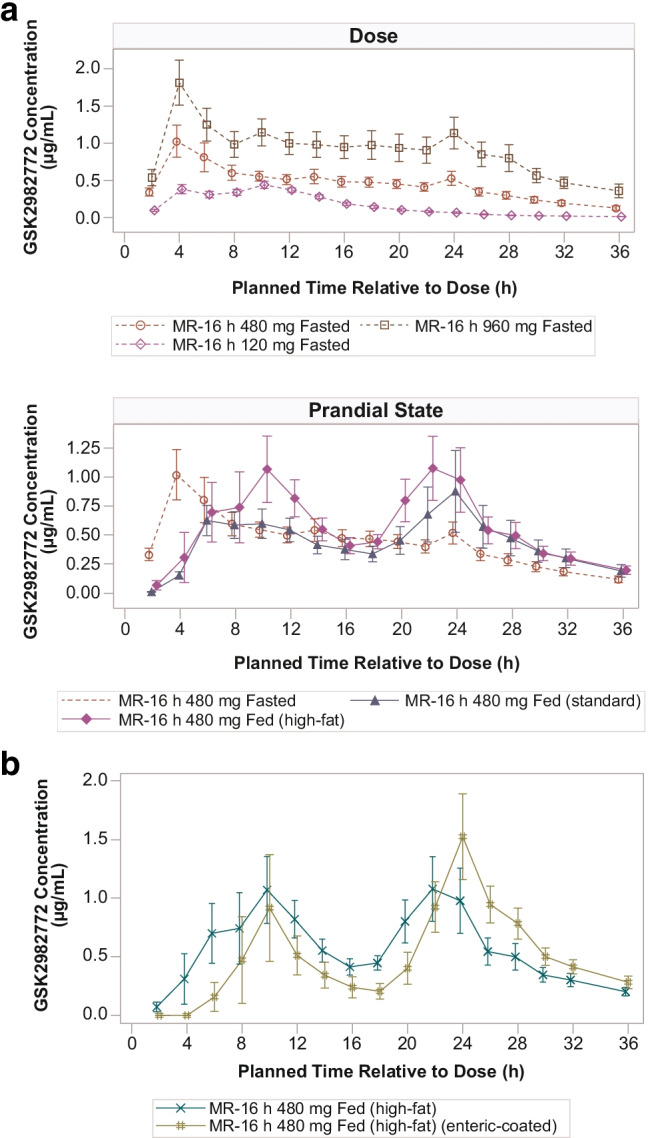
Mean plasma GSK2982772 concentration–time plots (Part B). **(a)** Concentration–Time by Dose and Prandial State. **(b)** DiffCORE MR-16 h 480 mg Fed (high-fat) and Enteric-Coated MR-16 h 480 mg Fed (high-fat) h, hours; MR-16 h, modified release with 80% release at 16 h. Error bars represent ± 1 standard error.

**Fig. 5:**
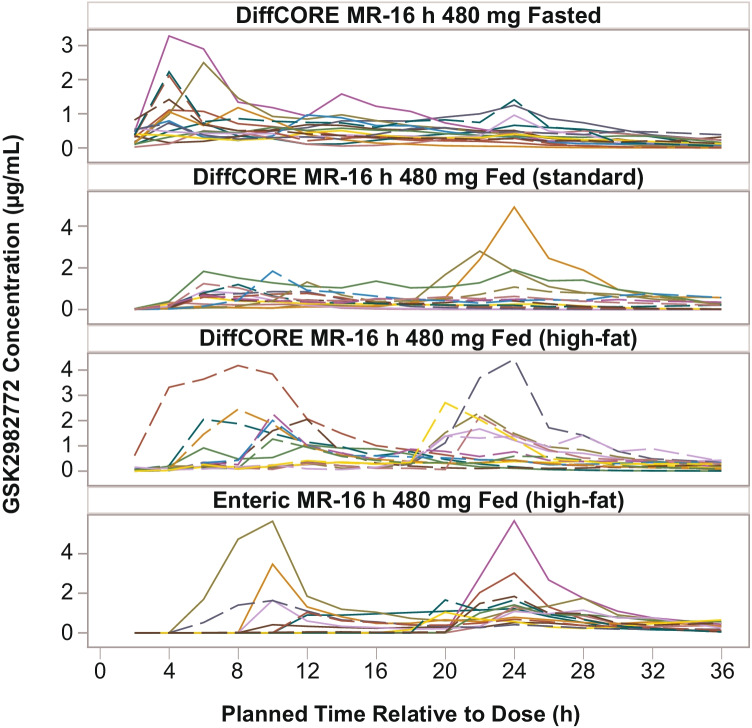
Individual subject GSK2982772 plasma concentration–time profiles for 480-mg MR-16 h dose. h, hours; MR-16 h, modified release with 80% release at 16 h.

Following administration of 480 mg MR-16 h with a standard meal, there was no overall impact on C_max_ or AUC_(0-inf)_ with geometric ratios of 1.02 and 0.97, relative to fasted state, respectively (Table II). Following administration of 480 mg MR-16 h with a high-fat meal, C_max_ and AUC were 1.73-fold and 1.42-fold higher, respectively, compared with the fasted state (Table II). Individual subjects’ concentration–time profiles showed that T_max_ was more variable when doses were administered with food and a greater proportion of subjects had a late peak when doses were administered with a high-fat meal compared with a standard meal (Fig. 5). When the 480 mg MR-16 h was administered with a high-fat meal, there were some subjects who had early and other subjects with late peaks, resulting in mean GSK2982772 plasma concentration–time profiles with apparent double peaking (Fig. 4a).

The mean GSK2982772 plasma concentration–time profile following administration of the fully enteric-coated MR-16 h with a high-fat meal was similar to that observed for DiffCORE MR-16 h with a high-fat meal (Fig. 4b). The number of subjects with early and late peaks after administration of enteric-coated MR-16 h with a high-fat meal was similar to the DiffCORE formulation with a high-fat meal. For the enteric MR-16 h formulation, C_max_ and AUC comparisons were similar to those observed for DiffCORE MR-16 h, with relative bioavailability from 0.890 to 0.975 (Table II). However, dosing of the enteric-coated MR-16 h led to a later appearance of GSK2982772 in the plasma (T_lag_ approximately 8 h) and a later median T_max_ of 22 h compared with 12 h for the MR-16 h DiffCORE formulation in the same prandial state.

## DISCUSSION

A previous prototype MR study identified a QD MM formulation, which had an *in vitro* release of 80% over 12 h. Although this formulation offered an acceptable QD profile in the fasted state, it was found to be susceptible to a food effect. When the MM formulation was administered with a high-fat meal, there was a 2.25-fold increase in C_max_ and 1.24-fold increase in AUC_(0-inf)_, and most of the exposure to GSK2982772 was observed within the first 12 h after dosing. Such a profile was considered not optimal for QD dosing. The current study built on results from the previous study and evaluated whether the GSK proprietary DiffCORE™ MR formulation could provide a QD profile in both the fed and fasted states.

The study was conducted using a proven drug development platform, Translational Pharmaceutics, which integrates GMP manufacturing and clinical testing to enable within-protocol decision making on formulation compositions, thereby reducing development time, cost, and risk (21). One of the conventional challenges when developing an MR formulation is to identify the *in vivo* release rate and dose required to achieve the target product profile. *In vitro* dissolution data are generated to describe formulation release; however, the assumption that this will be the same *in vivo* is unproven until clinical data are available. Often with MR formulations a reduction in overall exposure (AUC) is observed as when delivering to lower regions in the GI tract, where absorption is reduced. Contributing factors include reduced fluid volumes (for dissolution and solubilization), reduced surface area, and lower permeability. Similarly, there are recognized challenges and risks of using non-clinical models to inform MR formulation development due to significant inter-species anatomical and physiological differences (22). In the current study, flexibility was further maximized by inclusion of a two-dimensional formulation design space in the regulatory submission, which allowed quantitative changes in release rate (*via* controlling the ratio of two HPMC polymers) and dose during the clinical study to achieve the desired PK profile. The study design allowed multiple formulation iterations to be tested in the same individuals in both the fed and fasted state within a short time frame to allow efficient optimization of the GSK2982772 formulation.

Results from the previous prototype matrix formulation study were used to help pre-define the range of *in vitro* release rates of GSK2982772 to test in the current study. It was anticipated that in the fasted state, the DiffCORE technology tablet should exhibit similar behavior *in vivo* to the previously tested MM tablets, since both formulations had a similar core matrix formulation (13).

The plasma profiles and PK data from Part A demonstrated that the DiffCORE 240-mg tablets with 12–18 h *in vitro* release profiles provided the desired QD profile, but without the vulnerability to loss of performance when co-administered with a high-fat meal. This contrasts with the performance observed previously with minitablet and MM tablets (12). The improved performance after a high-fat meal with the DiffCORE formulation can be attributed to the suppression of tablet hydration and erosion whilst the enteric coat remains intact in the low pH environment of the stomach.

The plasma profiles and PK data from Part B, which explored the higher tablet strength of 480 mg MR-16 h, confirm the lack of a significant impact of prandial conditions on the performance of the selected formulation as a QD product, although some differences in T_max_ were observed between meal types. For example, T_max_ was delayed with the high-fat meal, which was likely due to calorie intake-related gastric emptying (23). The 1.73-fold increase in C_max_ and 1.42-fold increase in AUC when MR-16 h (480 mg) was co-administered with a high-fat meal was similar to that observed in the previous study for the MM-12 h (120 mg) formulation (2.25-fold for C_max_ and 1.24-fold for AUC). However, the extent of exposure to GSK2982772 for MR-16 h was generally equally distributed around T_max_ instead of most of the exposure to GSK2982772 being within the first 12 h after dosing as observed with the MM formulation. Therefore, the shape of the PK profile for the MR-16 h was consistent with a QD dosing regimen.

There was high inter-subject variability in the time to C_max_ when the 480-mg MR-16 h tablet was administered with a high-fat meal. For about half of the study subjects, T_max_ was reached before 12 h and, for the other half, T_max_ was reached after 12 h, resulting in an apparent double peak in the mean concentration–time profiles when administered with a high-fat meal, and to a lesser extent with a standard meal (Fig. 4). A plausible hypothesis is that the combination of a relatively large tablet, the co-administered high-fat meal, and subsequent food intake led to significant delays in gastric emptying in about half of the subjects. The high-fat meal regimen employed in this study is likely to represent a worst-case scenario in terms of the impact of food on the PK profile. Following the co-administration of DiffCORE MR with the high-fat breakfast, a midday meal was administered 4 h after the morning dose, followed by an evening meal 6 h later, and an evening snack 4 h after the evening meal. For some individuals, the time may have been inadequate to return to a fasted state and the associated fasted state motility (“housekeeper wave” associated with phase 3 motility) that would empty large, undissolved matrices from the stomach. Similar observations have been made in volunteers dosed with monitoring capsules (24). Despite this, the DiffCORE formulation is still acceptable for a QD dosing regimen because the concentration–time profiles on either side of C_max_ were generally flat. Individual plasma concentration–time profiles were flatter after 480 mg MR-16 h with a standard meal than after administration of 480 mg MR-16 h with a high-fat meal, suggesting that a standard meal has less impact on tablet retention in the stomach.

Despite GSK2982772 being a low-solubility drug, the DiffCORE MR-16 h formulation showed approximately dose-proportional increases in C_max_ and AUC_(0-inf)_ for 240 mg (Part A), 480 mg (Part B), and 960 mg (Part B). However, dose proportionality was not observed between 120 mg MR-16 h and the higher dose levels. Observed systemic exposure for 120 mg MR-16 h was higher than expected with the geometric mean C_max_ similar to 240 mg MR-16 h and AUC_(0-inf)_ approximately 75% that of 240 mg MR-16 h, instead of the expected 50%. This finding may be partly explained by additional lactose, used as a filler in the 120-mg MR-16 h tablet, which may have increased the release rate of GSK2982772.

One additional formulation was tested in Part B, in which the DiffCORE formulation was simplified into an enteric-coated MR formulation by omitting the holes in the coat from the manufacturing process. The performance of this formulation when dosed with a high-fat meal was similar to that of the DiffCORE formulation in terms of AUC and C_max_, however, quantifiable concentrations of GSK2982772 appeared later in the plasma (T_lag_ approximately 8 h) and median T_max_ was 22 h compared with 12 h for the MR-16 h DiffCORE formulation. This is expected, as the enteric coat is insoluble in media with a pH below ~ 6 and thus there will be no drug release in the stomach (25). The enteric coat, similar to the DiffCORE coat, dissolves in the higher pH of the intestinal tract and the drug is then released. In contrast, however, a proportion of the drug from the DiffCORE formulation is released from the drilled apertures in the enteric coat whilst the tablet is in the stomach, which explains why quantifiable concentrations of GSK2982772 were observed at the first PK sampling time point at 2 h post-dose. Even though the variability in PK profiles was similar for the enteric-coated and DiffCORE formulations, with a similar number of study subjects displaying early and late peaks, one theoretical advantage of the DiffCORE formulation over the simple enteric-coated formulation is the release of some drug in the stomach. Some of the individuals dosed with the enteric-coated formulation showed no quantifiable plasma concentrations of GSK2982772 until 18–20 h post-dose, consistent with a return to fasted-state motility overnight (Fig. 4). The slow release of drug through the drilled aperture whilst the DiffCORE MR formulation is still in the stomach may offer advantages over enteric-coated MR in terms of onset of action.

## CONCLUSIONS

Single doses of GSK2982772 DiffCORE MR and enteric-coated formulations were generally well tolerated in healthy subjects.

There was minimal impact of co-administration of a high-fat meal on the PK of GSK2982772 for the 240-mg MR-16 h tablet. Although administration of a high-fat meal resulted in higher C_max_ and AUC for the 480-mg MR-16 h tablet, the shape of the concentration–time curve was still consistent with a QD dosing regimen. Following a T_lag_ of approximately 8 h, C_max_ and AUC for the enteric-coated formulation were similar to DiffCORE MR-16 h when administered with a high-fat meal. The DiffCORE technology overcame the food effect vulnerability observed with the previous matrix MR formulation. The MR-16 h formulation was selected for further clinical development as a QD dosing regimen.

## Supplementary Information

Below is the link to the electronic supplementary material.

Supplementary file1 (DOCX 67 kb)

## Abbreviations

0-inf: Plasma concentration vs time curve from zero to infinity
AE: Adverse event
AUC: Area under the curve
BID: Twice daily
C_max_: Maximum plasma concentration
IR: Immediate release
MM: Matrix monolithic
MR: Modified release
MT: Minitablet
PK: Pharmacokinetic
QD: Once daily
RIPK1: Receptor-interacting protein kinase-1
TID: Thrice daily
T_max_: Time to maximum concentration
TNF: Tumor necrosis factor

## Declarations

### Data Sharing Statement

The datasets generated during and/or analysed during the current study are not publicly available. Within 6 months of this publication, anonymized individual subject data, the annotated case report form, protocol, reporting and analysis plan, data set specifications, raw dataset, analysis-ready dataset, and clinical study report will be available for research proposals approved by an independent review committee. Proposals should be submitted to www.clinicalstudydatarequest.com. A data access agreement will be required.
