# Supplementary material for: Development of a Once-Daily Modified-Release Formulation for the Short Half-Life RIPK1 Inhibitor GSK2982772 using DiffCORE Technology
Source: Pharm Res. 2022 Jan 5;39(1):153–65. doi: 10.1007/s11095-021-03124-7 (PMC8837545; doi:10.1007/s11095-021-03124-7)
Supplement: Supplementary file 1 — Supplementary file1 (DOCX 67 kb) [file 11095_2021_3124_MOESM1_ESM.docx]

***Pharmaceutical Research***

# Electronic Supplementary Material

**Development of a Once-Daily Modified-Release Formulation for the Short Half-Life RIPK1 Inhibitor GSK2982772 using DiffCORE Technology**

Debra Tompson^1^, Mark Whitaker^2^, Rennan Pan^3^, Geoffrey Johnson^4^, Teresa Fuller^5^, Vanessa Zann^6^, Litza McKenzie^6^, Kathy Abbott-Banner^7^, Simon Hawkins^7^, Marcy Powell^8^

^1^Clinical Pharmacology Modelling and Simulation, GlaxoSmithKline, Medicines Research Centre, Gunnels Wood Road, Stevenage, Hertfordshire SG1 2NY, UK

^2^Medicine Process Delivery, GlaxoSmithKline, Dave Jack Medicines Development Centre, Park Road, Ware, Hertfordshire SG12 0DP, UK

^3^Pharmaceutical Development, GlaxoSmithKline, 1250 S. Collegeville Road, Collegeville, PA 19426, USA

^4^Development Biostatistics, GlaxoSmithKline, 1250 S. Collegeville Road, Collegeville, PA 19426, USA

^5^Global Clinical Sciences and Delivery, GlaxoSmithKline, Medicines Research Centre, Gunnels Wood Road, Stevenage, Hertfordshire SG1 2NY, UK

^6^Quotient Sciences Limited, Mere Way, Ruddington, Nottingham NG11 6JS, UK

^7^GlaxoSmithKline, 980 Great West Road, Brentford, Middlesex TW8-9GS, UK

^8^Safety and Medical Governance, GlaxoSmithKline, 5 Moore Drive, Research Triangle Park, NC 27709-3398, USA

**Corresponding Author:**

*Debra J. Tompson, MSc

GlaxoSmithKline

Email: debra.j.tompson@gsk.com

**Online Resource I. Subject Demographics**

|  | **Part A**  (*N*=17) | **Part B**  (*N*=16) |
| --- | --- | --- |
| Age (years) |  |  |
| Mean (SD) | 43.3 (12.2) | 54.0 (6.3) |
| Sex, n (%) |  |  |
| Female | 7 (41) | 6 (38) |
| Male | 10 (59) | 10 (63) |
| BMI (kg/m^2^) |  |  |
| Mean (SD) | 25.7 (3.1) | 26.2 (3.2) |
| Height (cm) |  |  |
| Mean (SD) | 171 (11.6) | 171 (7.8) |
| Weight |  |  |
| Mean (SD) | 75.4 (13.7) | 76.8 (14.1) |
| Ethnicity, n (%) |  |  |
| Not Hispanic or Latino | 17 (100) | 16 (100) |
| Race, n (%) |  |  |
| African American/African heritage | 0 | 1 (6) |
| Asian – Central/South Asian heritage | 0 | 1 (6) |
| White – White/Caucasian/European heritage | 16 (94) | 13 (81) |
| Mixed race | 0 | 1 (6) |

SD, standard deviation.

**Online Resource II. Summary of Adverse Events**

| **Part A** | | | | | | |
| --- | --- | --- | --- | --- | --- | --- |
| **Preferred Term**  ***n* (%)** | **240-mg IR Fasted**  **(*N*=16)** | **240-mg MR-12 h Fasted**  **(*N*=16)** | **240-mg MR-12 h Fed (high-fat)**  **(*N*=12)** | **240-mg MR-16 h Fasted**  **(*N*=12)** | **240-mg MR-18 h Fasted**  **(*N*=16)** | **240-mg MR-18 h Fed (high-fat)**  **(*N*=16)** |
| Participants with any AE | 2 (13) | 5 (31) | 3 (25) | 4 (33) | 3 (19) | 3 (19) |
| Headache | 1 (6) | 2 (13) | 0 | 0 | 1 (6) | 2 (13) |
| Syncope | 0 | 0 | 0 | 0 | 0 | 1 (6) |
| Upper respiratory tract infection | 1 (6) | 1 (6) | 0 | 2 (17) | 0 | 0 |
| Gastroenteritis | 0 | 0 | 1 (8) | 0 | 0 | 1 (6) |
| Nasopharyngitis | 0 | 0 | 1 (8) | 1 (8) | 0 | 0 |
| Abdominal pain lower | 0 | 0 | 0 | 0 | 1 (6) | 0 |
| Vomiting | 0 | 1 (6) | 0 | 0 | 0 | 0 |
| Application site irritation | 0 | 0 | 0 | 0 | 1 (6) | 0 |
| Chest discomfort | 0 | 1 (6) | 0 | 0 | 0 | 0 |
| Transaminases increased | 0 | 0 | 0 | 0 | 0 | 1 (6) |
| Back pain | 0 | 0 | 1 (8) | 0 | 0 | 0 |
| Nasal congestion | 0 | 0 | 1 (8) | 0 | 0 | 0 |
| Rash | 0 | 1 (6) | 0 | 0 | 0 | 0 |
| Hematoma | 0 | 0 | 0 | 1 (8) | 0 | 0 |
| **Part B** | | | | | | |
| **Preferred Term**  ***n* (%)** | **480-mg MR-16 h Fasted**  **(*N*=16)** | **480-mg MR-16 h Fed (standard)**  **(*N*=14)** | **480-mg MR-16 h Fed (high-fat)**  **(*N*=15)** | **480-mg MR-16 h Fed (enteric coated)**  **(*N*=14)** | **960-mg MR-16 h Fasted**  **(*N*=15)** | **120-mg MR-16 h Fasted**  **(*N*=15)** |
| Participants with any AE | 8 (50) | 4 (29) | 1 (7) | 3 (21) | 3 (20) | 3 (20) |
| Headache | 3 (19) | 1 (7) | 0 | 1 (7) | 2 (13) | 0 |
| Nasopharyngitis | 0 | 0 | 1 (7) | 1 (7) | 0 | 0 |
| Oral herpes | 1(6) | 0 | 0 | 0 | 0 | 0 |
| Contusion | 0 | 1 (7) | 0 | 0 | 0 | 0 |
| Soft tissue injury | 0 | 0 | 0 | 1 (7) | 0 | 0 |
| Tooth fracture | 0 | 0 | 0 | 0 | 0 | 1 (7) |
| Rectal hemorrhage | 0 | 0 | 0 | 0 | 1 (7) | 0 |
| Tooth loss | 0 | 0 | 0 | 0 | 1 (7) | 0 |
| Vomiting | 0 | 0 | 0 | 0 | 1 (7) | 0 |
| Catheter site swelling | 0 | 0 | 0 | 0 | 0 | 1 (7) |
| Peripheral swelling | 0 | 0 | 0 | 1 (7) | 0 | 0 |
| Back pain | 0 | 1 (7) | 0 | 0 | 0 | 0 |
| Musculoskeletal stiffness | 0 | 0 | 0 | 0 | 0 | 1 (7) |
| Depressed mood | 1 (6) | 0 | 0 | 0 | 0 | 0 |
| Nightmare | 1 (6) | 0 | 0 | 0 | 0 | 0 |
| Actinic keratosis | 1 (6) | 0 | 0 | 0 | 0 | 0 |
| Rash | 0 | 0 | 0 | 0 | 1 (7) | 0 |
| Conjunctival hemorrhage | 1 (6) | 0 | 0 | 0 | 0 | 0 |
| Cardiac murmur | 0 | 1 (7) | 0 | 0 | 0 | 0 |

AE, adverse event.

Online Resource III. Summary statistics of derived plasma GSK2982772 PK parameters (geometric mean [95% CI]) by DiffCORE MR formulation/prandial state (Part A)

| **Treatment  (240 mg)** | **C_max_ (μg/mL)** | **T_max_ (h)^a^** | **AUC_(0-inf)_ (h·μg/mL)** | **C_24h_ (μg/mL)** |
| --- | --- | --- | --- | --- |
| MR-12 h fasted (*N*=16) | 0.682  (0.557–0.835) | 5.0  (4.0–12.0) | 7.76 (6.88–8.75) | 0.196  (0.143–0.269) |
| MR-12 h fed  (high-fat) (*N*=12) | 0.824 (0.619–1.10) | 8.0  (2.0–22.0) | 9.37 (7.68–11.4) | 0.213  (0.142–0.318) |
| MR-16 h fasted (*N*=12) | 0.466  (0.392–0.553) | 6.0  (4.0–16.0) | 7.08 (4.69–10.6) | 0.165  (0.098–0.278) |
| MR-18 h fasted (*N*=16) | 0.527  (0.463–0.599) | 10.0  (4.0–24.2) | 8.35 (7.08–9.84) | 0.211  (0.155–0.287) |
| MR-18 h fed (high-fat) (*N*=16) | 0.678  (0.535–0.861) | 11.0  (8.0–22.3) | 8.22 (6.54–10.3) | 0.265  (0.179–0.393) |

CI, confidence interval; h, hours; IR, intermediate release; MR-12 h, modified release with 80% release at 12 h; MR-16 h, modified release with 80% release at 16 h; MR-18 h, modified release with 80% release at 18 h.

^a^T_max_ presented as median (range).

**Online Resource IV. Summary statistics of derived plasma GSK2982772 PK parameters for DiffCORE and enteric-coated MR formulations (geometric mean [95% CI]) (Part B)**

| **Treatment** | **C_max_ (μg/mL)** | **T_max_ (h)^a^ Median  (min, max)** | **AUC_(0-inf)_ (hr∙μg/mL)** | **C_24h_ (μg/mL)** |
| --- | --- | --- | --- | --- |
| **PK Parameters by Dose** | | | | |
| 480 mg MR-16 h fasted (*N*=16) | 1.10  (0.807–1.50) | 6.00  (4.00–24.2) | 13.6 (11.367–16.275) | 0.401  (0.258–0.624) |
| 960 mg MR-16 h fasted (*N*=15) | 1.75  (1.23–2.49) | 4.00  (2.00–36.0) | 29.9  (20.6–43.3) | 0.853  (0.538–1.35) |
| 120 mg MR-16 h fasted (*N*=15) | 0.517  (0.434–0.616) | 10.00  (4.00–14.0) | 5.38  (4.52–6.40) | 0.051  (0.034–0.075) |
| **PK Parameters by Prandial State** | | | | |
| 480 mg MR-16 h fed (standard) (*N*=14) | 1.14 (0.767–1.68) | 9.00 (4.00–24.00) | 13.2 (8.10–21.4) | 0.278 (0.091–0.851) |
| 480 mg MR-16 h fed (high-fat) (*N*=15) | 1.90 (1.37–2.61) | 12.00 (6.00–28.00) | 20.1 (15.0–27.0) | 0.622 (0.356–1.09) |
| 480 mg MR-16 h fed (high-fat, enteric-coated) (*N*=14) | 1.79 (1.22–2.63) | 22.0 (10.0–24.1) | 20.2 (12.5–32.7) | 1.18 (0.782–1.77) |

CI, confidence interval; h, hours; MR-12 h, modified release with 80% release at 12 h; MR-16 h, modified release with 80% release at 16 h; MR-18 h, modified release with 80% release at 18 h.

^a^T_max_ presented as median (range).
